# Analysis of enterovirus genotypes in the cerebrospinal fluid of children associated with aseptic meningitis in Liaocheng, China, from 2018 to 2019

**DOI:** 10.1186/s12879-021-06112-9

**Published:** 2021-05-01

**Authors:** Jing Wang, Min Meng, Huan Xu, Ting Wang, Ying Liu, Han Yan, Peiman Liu, Daogang Qin, Qiaozhi Yang

**Affiliations:** Key Laboratory for Pediatrics of Integrated Traditional and Western Medicine, Liaocheng People’s Hospital, No. 67 Dongchang West Road, Liaocheng, 252000 Shandong Province PR China

**Keywords:** Enterovirus (EV), Aseptic meningitis, Phylogenetic analysis, Children, Liaocheng

## Abstract

**Background:**

Aseptic meningitis is most often caused by enteroviruses (EVs), but EVs associated with aseptic meningitis have not yet been reported in Liaocheng. The aim of this study was to determine the prevalence and genetic characteristics of EVs causing aseptic meningitis in children in Liaocheng.

**Methods:**

We reviewed the epidemiological and clinical characteristics of 504 paediatric cases of aseptic meningitis in Liaocheng from 2018 to 2019 and analysed the phylogeny of the predominant EV types causing this disease.

**Results:**

A total of 107 children were positive for EV in cerebrospinal fluid samples by nested PCR. Most of the positive patients were children 13 years old or younger and had symptoms such as fever, headache and vomiting (*P <* 0.05). The seasons with the highest prevalence of EV-positive cases were summer and autumn. The 107 EV sequences belonged to 8 serotypes, and echovirus types 18, 6 and 11 were the three dominant serotypes in Liaocheng during the 2-year study period. Phylogenetic analyses demonstrated that the E18 and E6 isolates belonged to subgenotype C2, while the E11 isolates belonged to subgenotype D5. VP1 analysis suggested that only one lineage of these three types was cocirculating in the Liaocheng region.

**Conclusions:**

This study demonstrated the diverse EV genotypes contributing to a large outbreak of aseptic meningitis in Liaocheng. Therefore, large-scale surveillance is required to assess the epidemiology of EVs associated with aseptic meningitis and is important for the diagnosis and treatment of aseptic meningitis in Liaocheng.

**Supplementary Information:**

The online version contains supplementary material available at 10.1186/s12879-021-06112-9.

## Introduction

Enteroviruses (EVs) are small, nonenveloped, positive single-stranded RNA viruses from the genus *Enterovirus* within the family *Picornaviridae* [1]*.* To date, 116 serotypes belonging to four EV species (EV-A, B, C, and D) have been shown to infect humans on the basis of neutralizing assays and sequence analysis of the major capsid VP1 gene [2]. EVs are predominantly spread from person to person via the faecal-oral route and infect approximately a billion people worldwide each year; they typically occur in the summer and autumn in restricted geographical areas or communities, leading to increased admissions to hospital wards for short periods [3]. They are ubiquitous and cause diverse clinical manifestations, ranging from mild presentations, including minor febrile illness, gastrointestinal diseases and rash, to more severe syndromes, such as acute flaccid paralysis, myocarditis, neonatal sepsis, acute haemorrhagic conjunctivitis, encephalitis and aseptic meningitis [4].

Aseptic meningitis, defined as the most commonly observed CNS infection in sterile cerebrospinal fluid (CSF), causes different clinical presentations according to the patient’s age and immune status [5, 6]. Various studies conducted over the past several decades have shown that most EV-B types are major causative agents of this disease, and numerous EV-B-associated meningitis outbreaks have been reported throughout the world [7–13]. Shandong is a coastal province in China. There have been outbreaks of aseptic meningitis caused by EV-Bs (e.g., E6, E30, CVB3 and CVB5) in central eastern Shandong Province [14–18]. Liaocheng City is located in the western region of Shandong Province, and no information on the EVs causing aseptic meningitis in children in the city has been reported. There is limited information on circulating EV serotypes and associated clinical phenotypes in the paediatric population in the Liaocheng region, and such knowledge is essential for laboratory diagnostics, patient management and future outbreak responses.

The main aim of this study was to investigate the genotypes of EVs contributing to aseptic meningitis and the associations between EV types, clinical manifestations and CSF laboratory findings in Liaocheng over the period 2018 to 2019. Furthermore, the intratypical genetic variation of three predominant EV types was assessed through nested RT-PCR and phylogenetic analysis targeting the VP1 region, directly in CSF samples of patients.

## Materials and methods

### Patients and clinical sample collection

Liaocheng City is located in the western region of Shandong Province between 35°47′ ~ 37°02′ north latitude and 115°16′ ~ 116°32′ east longitude. Liaocheng covers an area of 8715 km^2^, accounting for 5.5% of Shandong Province. This area has a population of 5,935,700, with a population density of 681.1 people per square kilometre.

A total of 504 CSF specimens from children admitted with suspected aseptic meningitis were collected in Liaocheng People’s Hospital (China) from 1 January 2018 to 31 December 2019. The CSF specimens were sent to our laboratory and were stored at approximately 4 °C during sample transport and then at - 80 °C until analysis. Data on patient demographics, clinical symptoms, and CSF laboratory findings were collected retrospectively from the patients’ clinical histories. All statistical tests were conducted using SPSS software version 18.0. Data were presented as the mean ± standard error (SE) for continuous variables, and a *P*-value of less than 0.05 was considered statistically significant (*P* < 0.05).

### Enterovirus diagnosis and molecular genotyping

Viral RNA was extracted from 200 μl of the CSF samples by using the MagBeads RNA Extraction Kit (Liferiver, China) according to the manufacturer’s recommended procedure. Species-specific nested primers were used to amplify the VP1 gene region from EV-A and EV-B based on the standard protocol [19].

Reverse transcription polymerase chain reaction (RT-PCR) and PCR were conducted using the SuperScript™ III OneStep RT-PCR System (Invitrogen, USA) and Pyrobest™ DNA Polymerase (TaKaRa, Japan). The first round of PCR was performed in a 25 μl reaction containing 8 μl RNA, 1 μl RT-PCR mix, 12.5 μl of 2× reaction buffer, and 20 pmol each of the outer primers for EV-A or EV-B. The amplification program was as follows: 50 °C for 30 min, followed by 30 cycles of a predenaturing stage at 94 °C for 2 min, denaturing stage at 94 °C for 30 s, annealing stage at 52 °C for 30 s and elongation stage at 72 °C for 1 min. Subsequently, the second round of PCR was performed with 2 μl of the first-round PCR products and 20 pmol of each of the inner primers in a volume of 25 μl under the same PCR conditions described above. The PCR product was collected following electrophoresis in a 1.0% agarose gel with ethidium bromide staining under UV illumination. The positive PCR products were purified and bidirectionally sequenced on an ABI3730XL automatic sequencer (Applied Biosystems, Foster City, CA, USA). The obtained sequences were used for molecular typing with the online Enterovirus Genotyping Tool version 0.1.

### Sequence analysis and phylogenetic analysis

The BLASTn web site was used to align multiple partial VP1 sequences (https://blast.ncbi.nlm.nih.gov/Blast.cgi). Nucleotide sequence alignment and homologous comparisons were performed using BioEdit software (version 7.0.5.3). Multiple alignments were generated with the Clustal W program. Phylogenetic analyses based on the VP1 sequences were performed in the MEGA 6 program using the neighbour-joining (NJ) method with maximum composite likelihood and Kimura 2-parameter models [20]. The details of the the sequences including in the phylogenetic analysis can be found in Additional file 1. Bootstrap testing with 1000 duplicates was used to test the strength of the phylogenetic trees. Bootstrap values > 70% were considered statistically significant for grouping. In addition, a nucleotide difference in the VP1 region of more than 15% between groups or 8% within groups was used to distinguish genotypes and subgenotypes.

### Nucleotide sequence accession number

The complete VP1 sequences of the EV strains obtained in this study were deposited in the GenBank database under the accession numbers MT901934-MT901942, MT950542-MT950636, and MT646123-MT646125.

## Results

### Cases and epidemiology

**Fig. 1:**
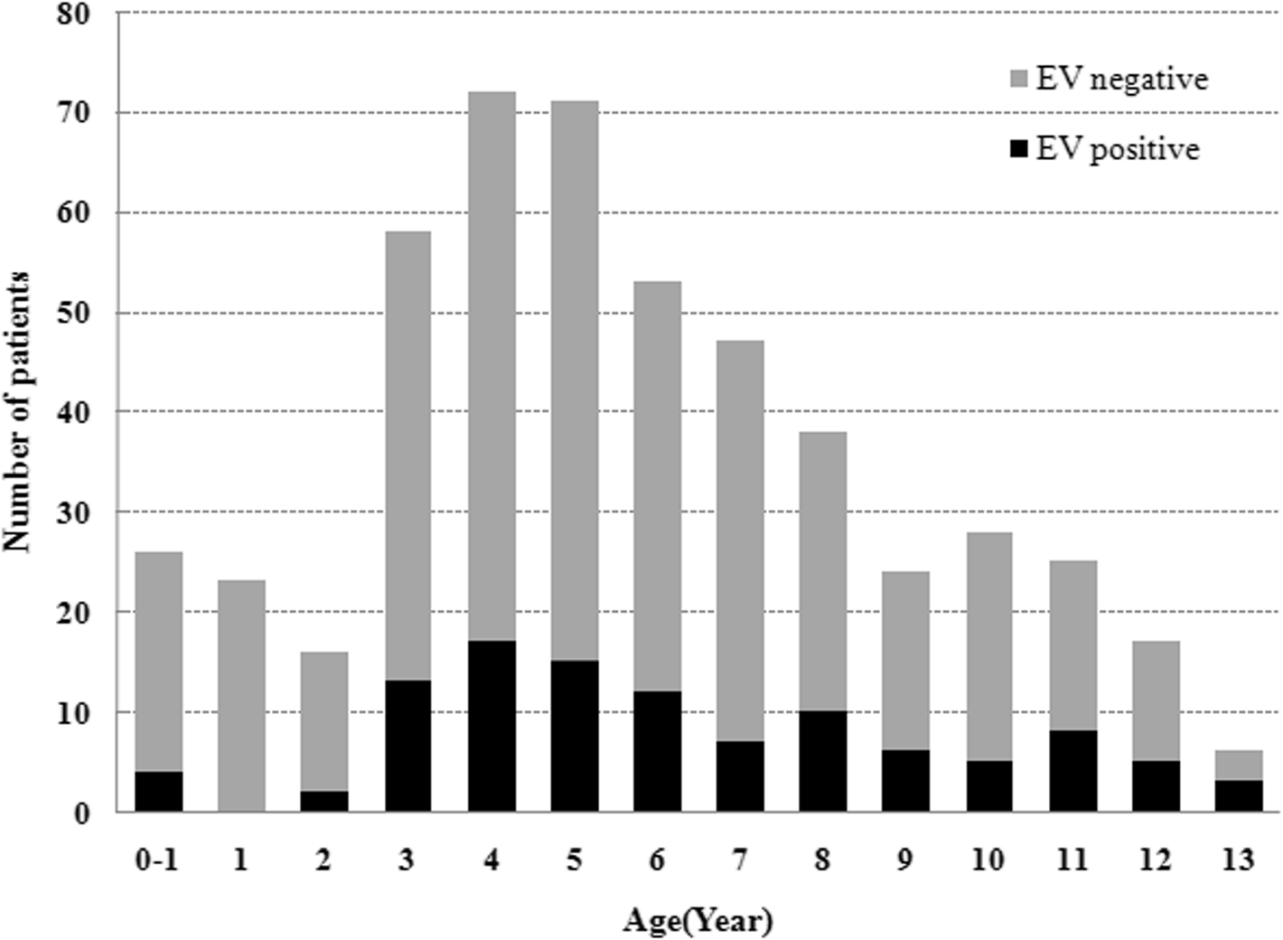
Age distribution of aseptic meningitis patients in Liaocheng (*n* = 504)

**Fig. 2:**
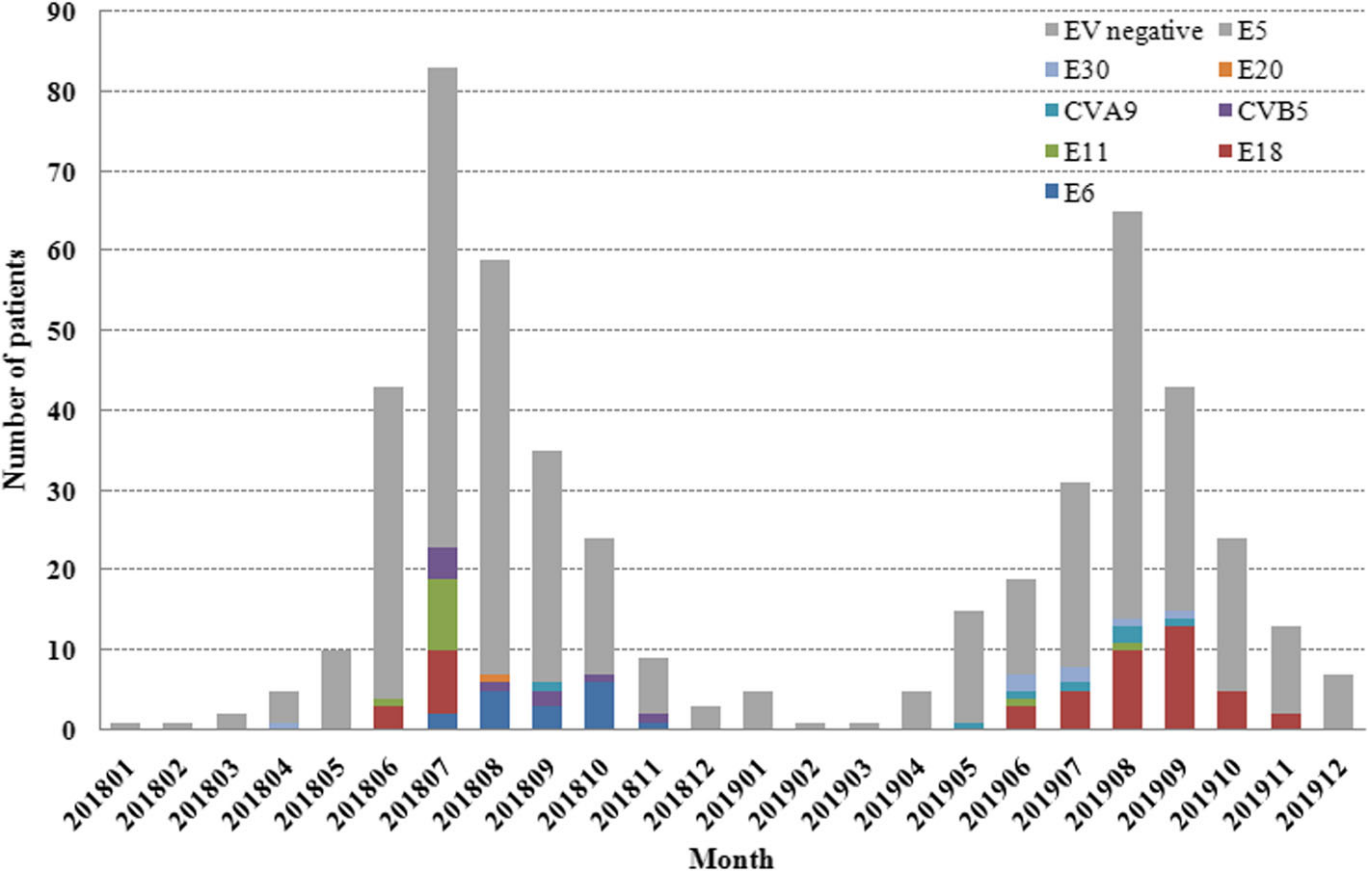
Monthly distribution of enterovirus genotypes identified in aseptic meningitis patients from 2018 to 2019, Liaocheng, China

A total of 504 hospitalized patients suspected of having aseptic meningitis were reported in the period from 2018 to 2019. Among these patients, 107 (21.2%) were positive for EVs by RT-PCR. Figure 1 shows the age distribution among all the patients. Among EV-positive patients, the ages ranged from 1 month to 13 years. The majority of EV cases (53.3%, 57/107) identified in this study occurred in children aged between three and six and were most frequently found in preschool children. The sex ratio was 1.97:1, corresponding to 71 male and 36 female cases. There was no significant difference in the male-to-female ratio (*P* > 0.05) between EV-positive patients and EV-negative patients. The analysis of the monthly distribution of patients showed that enteroviral infections were observed throughout the year, but 98.1% (105/107) of the cases occurred in summer and autumn (Fig. 2). In 2018, most of the HEV serotypes (98%, 49/50) detected were distributed from June to November (4 in June, 23 in July, 7 in August, 6 in September, 7 in October and 2 in November). Similarly, most of the EVs (98.2%, 56/57) were detected between June and November (7 in June, 9 in July, 16 in August, 17 in September, 5 in October and 2 in November) in 2019. Only 2 cases were detected between April and May from 2018 to 2019. Our results revealed that EV infection shows typical seasonal features in temperate climates.

### Clinical characteristics and laboratory findings

The clinical characteristics and laboratory findings of the EV-positive and EV-negative patients are shown in Table 1. The most common clinical symptoms of EV-positive patients were fever, headache and vomiting. Approximately 94.4% of the EV-positive patients suffered from fever at the time of hospital admission. There were no significant differences in the hospital stay or fever duration before admission between EV-positive patients and EV-negative patients (*P* > 0.05). The data showed that the frequency of headache and dizziness and neck stiffness was significantly higher in EV-positive patients than in EV-negative patients (*P* < 0.05). Almost half of the patients had complications such as neurologic, circulatory or respiratory system symptoms. CSF pleocytosis and protein levels were found to be significantly increased in the EV-positive group relative to the EV-negative group (*P* < 0.05). In general, EV was found to be statistically associated with pleocytosis, which was found 92.5% (99/107) of EV-positive cases, whereas 7.5% (8/107) of the EV cases did not involve pleocytosis.

**Table 1:** Comparison of clinical characteristics and laboratory findings between EV-positive patients and EV-negative patients from 2018 to 2019

Characterics	EV-positive patients (***n*** = 107)	EV-negative patients (***n*** = 397)	***P*** value
Age^a^ (years)	6.6 ± 3.1	5.6 ± 3.1	0.019
Male [no. (%)]	71(66.4)	258(65)	0.792
Hospital stay^a^ (days)	9.3 ± 2.3	9.2 ± 2.2	0.902
Fever duration before admission^a^ (days)	2.3 ± 2.4	2.9 ± 2.8	0.059
Clinical symptoms and signs [no. (%)]			
Fever	101(94.4)	366(92.2)	0.438
Headache	98(91.6)	304(76.6)	0.001
Vomiting	47(44)	185(46.6)	0.622
Lethargy	44(41.1)	129(32.5)	0.095
dizzy	56(52.3)	148(37.3)	0.005
Convulsion	1(0.9)	28(7.1)	0.016
Neck stiffness	19(17.8)	30(7.6)	0.002
Anterior fontanelle bulge	0(0)	4(1)	0.297
Babinski’s sign and/or Brudzinski’s sign	19(17.8)	116(29.2)	0.017
Respiratory symptoms^b^	78(72.9)	239(60.2)	0.017
CSF findings [no. (%)]			
White blood cell > 10 × 10^6^/L	99(92.5)	275(69.3)	< 0.001
Protein > 40 mg/dL	21(19.6)	37(9.3)	0.003

a mean ± SD

^b^ Respiratory symptoms include coarse breath sounds, cough and other symptoms

### Enterovirus genotypes

Among the 504 specimens, 107 (21.2%) were positive for EVs by RT-PCR, which were successfully serotyped by sequencing the amplicons of the VP1 sequence. A total of 8 serotypes were detected, with different detection rates: E6 (15.9%, 17/107), E18 (45.8%, 49/107), E11 (11.2%, 12/107), CVB5 (8.4%, 9/107), CVA9 (6.5%, 7/107), E30 (6.5%, 7/107), E5 (4.7%, 5/107), and E20 (1%, 1/107). All of the serotypes were belonged to the EV-B species. The EV positivity rate was 18.2% (50/275) in 2018 and 24.9% (57/229) in 2019. The proportion of predominant pathogens varied significantly between years, as did the proportion of each serotype. The predominant serotype changed from E6 (34%, 17/50) in 2018 to E18 (66.7%, 38/57) in 2019. Genotypes E18, E6, and E11 were the three predominant types throughout 2018 to 2019.

### Phylogenetic analysis of EV genotypes

Phylogenetic analysis and homologous comparisons were conducted using the complete VP1 sequences of 49 E18, 17 E6 and 12 E11 isolates obtained in this study and the global reference sequences of 43 E18, 59 E6 and 75 E11 isolates retrieved from GenBank, which were representatives of different genotypes and subgenotypes.

**Fig. 3:**
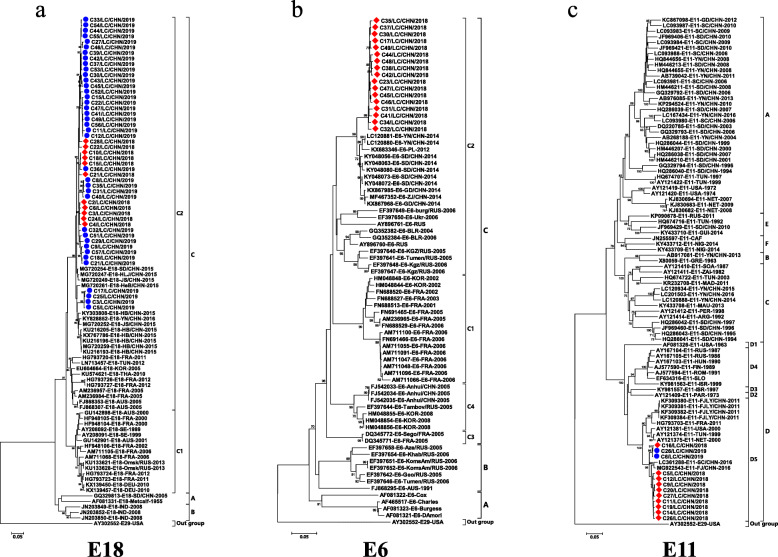
Phylogenetic analyses based on VP1 sequences of E18 (panel **a**), E6 (panel **b**) and E11 (panel **c**) causing aseptic meningitis in Liaocheng. Phylogenetic trees were constructed on the basis of partial VP1 gene sequences using neighbour-joining methods. Red diamonds and blue circles indicate isolates obtained in this study. The prototype E29-USA strain was used as an outgroup

All Liaocheng E18 isolates were classified within the C2 subgenotype, along with meningitis-related E18 isolates from the countries of China, France, Tunisia, Korea, Thailand and Australia. E18-314, a previous reference strain causing an aseptic meningitis outbreak in Hebei in 2015, also belonged to this subgenotype. Homologous analysis revealed that the isolates shared 94.6-96.2% nucleotide similarity with the meningitis-related E18 strain from Hebei Province (Fig. 3a).

Similarly, 17 E6 VP1 sequences from Liaocheng fell belonged to the C2 subtype, along with isolates from different areas of China, Poland, Russia and Belarus. Shandong E6 meningitis isolates obtained in 2014 also clustered in this subgenotype. Liaocheng E6 showed a closer relationship with the Shandong meningitis isolates, showing 95.7-96.7% similarity (Fig. 3b).

Additionally, all Liaocheng E11 VP1 sequences were grouped into subgenotype D5, along with earlier Chinese isolates and foreign isolates from Tunisia, New Zealand, the United States, France and Russia. The E11 strains causing meningitis and hand, foot, and mouth disease (HFMD) in Fujian and Sichuan Provinces of China in 2011, 2016 and 2017 were also classified into subgenotype D5. Liaocheng E11 shared 91.1-98.5% VP1 nucleotide similarity with these reference strains (Fig. 3c).

## Discussion

Aseptic meningitis is the most common CNS infection in which the CSF is negative for bacteria, and EVs, human parechovirus (HPeV), varicella zoster virus (VZV) and herpes simplex viruses (HSVs) 1 and 2 are the most common viral aetiologies [21, 22]. In particular, EVs are widely recognized as the main causal agents of aseptic meningitis, an association proven by virus genotyping in CSF specimens from both children and adults [23]. Various studies conducted over several decades have shown that children are the primary victims of this disease, and numerous EV meningitis outbreaks associated with EVs have been described [23–26]. Although the surveillance system for population-based EVs in mainland China is limited, related studies of EV meningitis outbreaks have been frequently reported in the provinces of Jiangsu [27], Gansu [28], Anhui [29], Zhejiang [30–32], Guangdong [8, 33, 34], Yunnan [10], Hebei [35] and Shandong [14–18, 36] in recent years. These outbreaks were characterized by a large number of hospitalized children, which is currently a pressing public health issue in China. Hence, rapid EV identification and genotyping in CSF samples are critical for investigating EV circulation and understanding the social burden of EV infection.

In this study, we described the molecular epidemiology and genotypes of EVs isolated from CSF samples from children hospitalized for aseptic meningitis in Liaocheng, China. Our results showed that 107 out of 504 (21.2%) examined cases of aseptic meningitis were positive for EVs and that 8 different EV genotypes were responsible for EV meningitis. Several previous studies showed that the identification rate of EV types from CSF ranged from 19.5 to 54.1% using previously developed assays [37–40]. The findings of our study were consistent with these studies, and the success rate of direct EV typing was low (21.2%). This could be attributed to the low viral load of CSF samples, the high variability of the VP1 region or the presence of other viral infections. The clinical signs and symptoms of enteroviral meningitis in children are mostly nonspecific; fever and headache were the most common symptoms in this study, followed by vomiting. Although EV-positive patients and EV-negative patients exhibited different clinical symptoms, these symptoms were not sufficient to distinguish between different aetiologies. During the 2-year period, the predominant EV types were E18 (45.8%), E6 (23.8%) and E11 (20.5%), which are most commonly associated with aseptic meningitis.

Phylogenetic analyses of the VP1 gene, as conducted in the present study, have been widely used for typing EVs and in molecular epidemiological investigations. Some EV serotypes (e.g., E6, E11, E18, E30 and CVB5) are still causing global epidemic outbreaks, while others (e.g., CVA9, E5 and E20) are primarily associated with endemic infections [18, 41–45]. E18 has caused many aseptic meningitis outbreaks in Germany, the United States, Japan, Korea, and other countries or regions [44, 46–50]. However, in mainland China, E18 meningitis has been less frequently reported. In China, an E18 encephalitis/meningitis outbreak was first reported in Hebei Province in 2015 [35]. Subsequently, E18-associated aseptic meningitis occurred in Zhejiang Province in 2014 and 2017 [32]. Previous studies showed that all E18 strains segregate into three genotypes: A, B, and C. Genotype C can be further divided into subgenotypes C1 and C2 [35]. Genotype A and subgenotype C2 circulate in mainland China, and subgenotype C2 has been the absolute dominant subgenotype in mainland China in recent years [35, 51]. In this study, all Liaocheng E18 isolates were found to belong to subgenotype C2. Chen et al. reported that the meningitis outbreak in Hebei Province was caused by a new C2 E18 strain, and our study supported this observation. Liaocheng E18 shows an evolutionarily close relationship with the reference E18-314 strain circulating in neighbouring Hebei Province, which suggests the formation of an exclusive transmission chain by E18-314 in China. As frequent travel and the return of migrant workers to Hebei Province might increase the important of E18, continuous surveillance is needed.

In China, E6 was first reported to be associated with an aseptic meningitis outbreak in 2005 [29]. Thereafter, E6 meningitis outbreaks occurred in Zhejiang Province in 2014 and 2017 [32]. In Shandong, sporadic E6 cases have occasionally been observed, and environmental surveillance of sewage proved that E6 was the predominant serotype in certain years [15, 16, 52, 53]. Furthermore, E6 was one of the predominant types responsible for EV meningitis in 2014 [18]. Previous studies showed that all known E6 strains could be divided into three genotypes, A, B and C. Genotype C could be subdivided into four subgenotypes, C1 to C4 [29]. Phylogenetic analyses showed that subgenotype C2 became the most frequently detected subgenotype in mainland China in recent years [18]. The Liaocheng E6 isolates belong to the C2 subgenotype, which is most closely related to the meningitis strains isolated in 2014 in Shandong Province, suggesting far less variety and continuous circulation in Shandong Province.

Echovirus 11 (E11) is one of the most common causes of meningitis in Russia, America, India, Japan and Israel, but meningitis outbreaks of E11 have not been found in mainland China [54–57]. Although no E11 meningitis outbreaks have been reported in China, EV surveillance has been performed, and cases in which E11 is associated with meningitis, acute flaccid paralysis (AFP) and HFMD have been identified in sewage or CSF samples from the provinces of Shandong, Fujian, Yunnan [42, 58, 59]. In this study, phylogenetic analysis revealed that Liaocheng E11 was grouped into subgenotype D5. In a previous worldwide epidemiological study on E11, genotype A was the predominant genotype found in mainland China, and D5 was the predominant subgenotype circulating in America, Europe and Russia [60]. However, E11 meningitis caused by subgenotype D5 was reported in Fujian Province in China in 2011, and E11 isolated under the HFMD surveillance system in China in 2016-2017 was also classified into subgenotype D5 [42, 59]. In this study, no Liaocheng E11 isolates were found to belong to genogroup A, and a great number of subgenotype D5 were identified, suggesting that the meningitis-related E11 isolates circulating in China in recent years mainly belong to this subgenotype.

There are some limitations to this study. First, the study was conducted in a tertiary hospital, and the number of cases was small. Second, the findings are limited by the use of nested RT-PCR performed for the detection of EVs in CSF samples, the low viral load of the samples, and the absence of testing for other viral infections in our study. In the future, to further elucidate the epidemiology of the pathogens responsible for aseptic meningitis, larger prospective multicentric studies will be developed, and other pathogens, such as HPeV, VZV, HSV-1 and HSV-2, should be considered and evaluated.

## Conclusions

Our study shows that EVs are common aetiological agents of aseptic meningitis among children in Liaocheng, China. Furthermore, this study reveals the circulation of eight meningitis-related EV genotypes in Liaocheng. E18, E6 and E11 are the three predominant genotypes. The subgenotype of E18 and E6 is C2, and the subgenotype of E11 is D5. Phylogenetic analyses of these three types revealed a single lineage circulating in the Liaocheng region, and they all show high genetic diversity relative to foreign isolates. Further surveillance of EV genotypes is needed to understand their changing genetic characteristics and clinical pathogenicity and to optimize the selection of interventions.

## Supplementary Information

**Additional file 1.** The sequences of EVs in this study.

## Data Availability

The datasets used and/or analysed during the current study are available from the corresponding author on reasonable request.

## Abbreviations

CSF: Cerebrospinal fluid
EV: Enterovirus
E5: Echovirus 5
E6: Echovirus 6
E11: Echovirus 11
E18: Echovirus 18
E20: Echovirus 20
E30: Echovirus 30
CVA9: Coxsackie virus A9
CVB5: Coxsackie virus B5
RT-PCR: Reverse transcription polymerase chain reaction
VP1: Viral capsid protein 1
HPeV: Human parechovirus
VZV: Varicella zoster virus
HSV1: Herpes simplex virus 1
HSV2: Herpes simplex virus 2
HFMD: Hand, foot, and mouth disease
AFP: Acute flaccid paralysis

## References

[1] Pons-Salort M, Parker EP, Grassly NC (2015). The epidemiology of non-polio enteroviruses: recent advances and outstanding questions. Curr Opin Infect Dis.

[2] Walker PJ, Siddell SG, Lefkowitz EJ, Mushegian AR, Dempsey DM, Dutilh BE, Harrach B, Harrison RL, Hendrickson RC, Junglen S, Knowles NJ, Kropinski AM, Krupovic M, Kuhn JH, Nibert M, Rubino L, Sabanadzovic S, Simmonds P, Varsani A, Zerbini FM, Davison AJ (2019). Changes to virus taxonomy and the international code of virus classification and nomenclature ratified by the international committee on taxonomy of viruses (2019). Arch Virol.

[3] Jubelt B, Lipton HL (2014). Enterovirus/picornavirus infections. Handb Clin Neurol.

[4] Nikonov OS, Chernykh ES, Garber MB, Nikonova EY (2017). Enteroviruses: classification, diseases they cause, and approaches to development of antiviral drugs. Biochemistry (Mosc).

[5] Martin NG, Iro MA, Sadarangani M, Goldacre R, Pollard AJ, Goldacre MJ (2016). Hospital admissions for viral meningitis in children in England over five decades: a population-based observational study. Lancet Infect Dis.

[6] Tattevin P, Tchamgoué S, Belem A, Bénézit F, Pronier C, Revest M (2019). Aseptic meningitis. Rev Neurol (Paris).

[7] Oberste MS, Maher K, Kennett ML, Campbell JJ, Carpenter MS, Schnurr D, Pallansch MA (1999). Molecular epidemiology and genetic diversity of echovirus type 30 (E30): genotypes correlate with temporal dynamics of E30 isolation. J Clin Microbiol.

[8] Xiao H, Guan D, Chen R, Chen P, Monagin C, Li W, Su J, Ma C, Zhang W, Ke C (2013). Molecular characterization of echovirus 30-associated outbreak of aseptic meningitis in Guangdong in 2012. Virol J.

[9] Holmes CW, Koo SS, Osman H, Wilson S, Xerry J, Gallimore CI (2016). Predominance of enterovirus B and echovirus 30 as cause of viral meningitis in a UK population. J Clin Virol.

[10] Zhu Y, Zhou X, Liu J, Xia L, Pan Y, Chen J, Luo N, Yin J, Ma S (2016). Molecular identification of human enteroviruses associated with aseptic meningitis in Yunnan province. Southwest China Springerplus.

[11] Smuts H, Cronje S, Thomas J, Brink D, Korsman S, Hardie D (2018). Molecular characterization of an outbreak of enterovirus-associated meningitis in Mossel Bay, South Africa, December 2015-January 2016. BMC Infect Dis.

[12] Miyoshi M, Goto A, Komagome R, Yamaguchi H, Maruo Y, Nakanishi M, Ishida S, Nagano H, Sugisawa T, Okano M (2020). Genetic characterization of a novel recombinant echovirus 30 strain causing a regional epidemic of aseptic meningitis in Hokkaido, Japan, 2017. Arch Virol.

[13] Ramalho E, Sousa I, Burlandy F, Costa E, Dias A, Serrano R, Oliveira M, Lopes R, Debur M, Burger M, Riediger I, Oliveira ML, Nascimento O, da Silva EE (2019). Identification and phylogenetic characterization of human enteroviruses isolated from cases of aseptic meningitis in Brazil, 2013-2017. Viruses..

[14] Wang HY, Xu AQ, Zhu Z, Li Y, Ji F, Zhang Y, Zhang L, Xu WB (2006). The genetic characterization and molecular evolution of echovirus 30 during outbreaks of aseptic meningitis. Zhonghua Liu Xing Bing Xue Za Zhi.

[15] Tao Z, Song Y, Wang H, Zhang Y, Yoshida H, Ji S, Xu A, Song L, Liu Y, Cui N, Ji F, Li Y, Chen P, Xu W (2012). Intercity spread of echovirus 6 in Shandong Province, China: application of environmental surveillance in tracing circulating enteroviruses. Appl Environ Microbiol.

[16] Tao Z, Song Y, Li Y, Liu Y, Jiang P, Lin X, Liu G, Song L, Wang H, Xu A (2012). Coxsackievirus B3, Shandong Province, China, 1990-2010. Emerg Infect Dis.

[17] Chen P, Tao Z, Song Y, Liu G, Wang H, Liu Y, Song L, Li Y, Lin X, Cui N, Xu A (2013). A coxsackievirus B5-associated aseptic meningitis outbreak in Shandong Province, China in 2009. J Med Virol.

[18] Chen P, Lin X, Liu G, Wang S, Song L, Tao Z, Xu A (2018). Analysis of enterovirus types in patients with symptoms of aseptic meningitis in 2014 in Shandong, China. Virology.

[19] Leitch EC, Harvala H, Robertson I, Ubillos I, Templeton K, Simmonds P (2009). Direct identification of human enterovirus serotypes in cerebrospinal fluid by amplification and sequencing of the VP1 region. J Clin Virol.

[20] Stecher G, Liu L, Sanderford M, Peterson D, Tamura K, Kumar S (2014). MEGA-MD: molecular evolutionary genetics analysis software with mutational diagnosis of amino acid variation. Bioinformatics..

[21] Putz K, Hayani K, Zar FA (2013). Meningitis. Prim Care.

[22] Wright WF, Pinto CN, Palisoc K, Baghli S (2019). Viral (aseptic) meningitis: a review. J Neurol Sci.

[23] Rudolph H, Schroten H, Tenenbaum T (2016). Enterovirus infections of the central nervous system in children: an update. Pediatr Infect Dis J.

[24] Zakhour R, Aguilera E, Hasbun R, Wootton SH (2018). Risk classification for enteroviral infection in children with meningitis and negative gram stain. Pediatr Emerg Care.

[25] Ai J, Xie Z, Liu G, Chen Z, Yang Y, Li Y, Chen J, Zheng G, Shen K (2017). Etiology and prognosis of acute viral encephalitis and meningitis in Chinese children: a multicentre prospective study. BMC Infect Dis.

[26] Hasbun R, Wootton SH, Rosenthal N, Balada-Llasat JM, Chung J, Duff S, Bozzette S, Zimmer L, Ginocchio CC (2019). Epidemiology of meningitis and encephalitis in infants and children in the United States, 2011-2014. Pediatr Infect Dis J.

[27] Zhao YN, Jiang QW, Jiang RJ, Chen L, Perlin DS (2005). Echovirus 30, Jiangsu Province, China. Emerg Infect Dis.

[28] Cui A, Yu D, Zhu Z, Meng L, Li H, Liu J, Liu G, Mao N, Xu W (2010). An outbreak of aseptic meningitis caused by coxsackievirus A9 in Gansu, the People's Republic of China. Virol J.

[29] Mao N, Zhao L, Zhu Z, Chen X, Zhou S, Zhang Y, Cui A, Ji Y, Xu S, Xu WB (2010). An aseptic meningitis outbreak caused by echovirus 6 in Anhui province, China. J Med Virol.

[30] Yan JY, Lu YY, Xu CP, Yu Z, Gong LM, Chen Y, Zhang YJ (2011). Study on the etiological and molecular characteristics of aseptic meningitis epidemic in Zhejiang Province in 2002-2004. Bing Du Xue Bao.

[31] Zheng S, Ye H, Yan J, Xie G, Cui D, Yu F, Wang Y, Yang X, Zhou F, Zhang Y, Tian X, Chen Y (2016). Laboratory diagnosis and genetic analysis of a family clustering outbreak of aseptic meningitis due to echovirus 30. Pathog Glob Health.

[32] Sun Y, Miao Z, Yan J, Gong L, Chen Y, Chen Y, Mao HY, Zhang YJ (2019). Sero-molecular epidemiology of enterovirus-associated encephalitis in Zhejiang Province, China, from 2014 to 2017. Int J Infect Dis.

[33] Xiao H, Huang K, Li L, Wu X, Zheng L, Wan C, Zhao W, Ke C, Zhang B (2014). Complete genome sequence analysis of human echovirus 30 isolated during a large outbreak in Guangdong Province of China, in 2012. Arch Virol.

[34] Lu J, Zheng H, Guo X, Zhang Y, Li H, Liu L, Zeng H, Fang L, Mo Y, Yoshida H, Yi L, Liu T, Rutherford S, Xu W, Ke C (2015). Elucidation of echovirus 30's origin and transmission during the 2012 aseptic meningitis outbreak in Guangdong, China, through continuing environmental surveillance. Appl Environ Microbiol.

[35] Chen X, Li J, Guo J, Xu W, Sun S, Xie Z (2017). An outbreak of echovirus 18 encephalitis/meningitis in children in Hebei Province, China, 2015. Emerg Microbes Infect.

[36] Wan YZ, Yue YY, Li P, Li ZH, Li J, Meng H (2007). Molecula-biological identification of pathogens which caused an outbreak of viral encephalitis in Jinan area. Zhonghua Shi Yan He Lin Chuang Bing Du Xue Za Zhi.

[37] Mirand A, Henquell C, Archimbaud C, Chambon M, Charbonne F, Peigue-Lafeuille H, Bailly JL (2008). Prospective identification of enteroviruses involved in meningitis in 2006 through direct genotyping in cerebrospinal fluid. J Clin Microbiol.

[38] Kadambari S, Bukasa A, Okike IO, Pebody R, Brown D, Gallimore C, Xerry J, Sharland M, Ladhani SN (2014). Enterovirus infections in England and Wales, 2000-2011: the impact of increased molecular diagnostics. Clin Microbiol Infect.

[39] Krasota A, Loginovskih N, Ivanova O, Lipskaya G (2016). Direct identification of enteroviruses in cerebrospinal fluid of patients with suspected meningitis by nested PCR amplification. Viruses..

[40] Dumaidi K, Al-Jawabreh A (2017). Molecular detection and genotyping of enteroviruses from CSF samples of patients with suspected sepsis-like illness and/or aseptic meningitis from 2012 to 2015 in West Bank, Palestine. PLoS One.

[41] Graf J, Hartmann CJ, Lehmann HC, Otto C, Adams O, Karenfort M, Schneider C, Ruprecht K, Bosse HM, Diedrich S, Böttcher S, Schnitzler A, Hartung HP, Aktas O, Albrecht P (2019). Meningitis gone viral: description of the echovirus wave 2013 in Germany. BMC Infect Dis.

[42] Li J, Yan D, Chen L, Zhang Y, Song Y, Zhu S, Ji T, Zhou W, Gan F, Wang X, Hong M, Guan L, Shi Y, Wu G, Xu W (2019). Multiple genotypes of echovirus 11 circulated in mainland China between 1994 and 2017. Sci Rep.

[43] Lema C, Torres C, Van der Sanden S, Cisterna D, Freire MC, Gómez RM (2019). Global phylodynamics of echovirus 30 revealed differential behavior among viral lineages. Virology..

[44] Rodà D, Pérez-Martínez E, Cabrerizo M, Trallero G, Martínez-Planas A, Luaces C, García-García JJ, Muñoz-Almagro C, Launes C (2015). Clinical characteristics and molecular epidemiology of enterovirus infection in infants < 3 months in a referral paediatric hospital of Barcelona. Eur J Pediatr.

[45] Faleye TOC, Adewumi OM, Klapsa D, Majumdar M, Martin J, Adeniji JA (2019). Genome sequences of two dual-serotype-specific echovirus 20 strains from Nigeria. Microbiol Resour Announc.

[46] Turabelidze G, Lin M, Butler C, Fick F, Russo T (2009). Outbreak of echovirus 18 meningitis in a rural Missouri community. Mo Med.

[47] Krumbholz A, Egerer R, Braun H, Schmidtke M, Rimek D, Kroh C (2016). Analysis of an echovirus 18 outbreak in Thuringia. Germany: insights into the molecular epidemiology and evolution of several enterovirus species B members.

[48] Kusuhara K, Saito M, Sasaki Y, Hikino S, Taguchi T, Suita S, Hayashi J, Wakatsuki K, Hara T (2008). An echovirus type 18 outbreak in a neonatal intensive care unit. Eur J Pediatr.

[49] Park K, Yeo S, Baek K, Cheon D, Choi Y, Park J, Lee SJ (2011). Molecular characterization and antiviral activity test of common drugs against echovirus 18 isolated in Korea. Virol J.

[50] Tsai HP, Huang SW, Wu FL, Kuo PH, Wang SM, Liu CC, Su IJ, Wang JR (2011). An echovirus 18-associated outbreak of aseptic meningitis in Taiwan: epidemiology and diagnostic and genetic aspects. J Med Microbiol.

[51] Chen X, Ji T, Guo J, Wang W, Xu W, Xie Z (2019). Molecular epidemiology of echovirus18 circulating in mainland China from 2015 to 2016. Virol Sin.

[52] Tao Z, Wang H, Li Y, Xu A, Zhang Y, Song L, Yoshida H, Xu Q, Yang J, Zhang Y, Liu Y, Feng L, Xu W (2011). Cocirculation of two transmission lineages of echovirus 6 in Jinan, China, as revealed by environmental surveillance and sequence analysis. Appl Environ Microbiol.

[53] Tao Z, Wang H, Li Y, Liu G, Xu A, Lin X, Song L, Ji F, Wang S, Cui N, Song Y (2014). Molecular epidemiology of human enterovirus associated with aseptic meningitis in Shandong Province, China, 2006-2012. PLoS One.

[54] Miller DG, Gabrielson MO, Bart KJ, Opton EM, Horstmann DM (1968). An epidemic of aseptic meningitis, primarily among infants, caused by echovirus 11-prime. Pediatrics..

[55] Patel JR, Daniel J, Mathan VI (1985). An epidemic of acute diarrhoea in rural southern India associated with echovirus type 11 infection. J Hyg (Lond).

[56] Miwa C, Sawatari S (1994). Epidemic of echo 11 virus infection in Gifu prefecture in 1993. Kansenshogaku Zasshi.

[57] Somekh E, Shohat T, Handsher R, Serour F (2001). An outbreak of echovirus 11 in a children's home. Epidemiol Infect.

[58] Yang J, Cui N, Wang H, Tao Z, Liu Y, Zhang H, Yoshida H, Song Y, Zhang Y, Song L, Li Y, Lin X, Ji S, Xu W, Xu A (2012). Evaluating the prevalence and molecular epidemiology of echovirus 11 isolated from sewage in Shandong Province, China in 2010. Virus Genes.

[59] Chen Q, Cao C, Zhang Y, He C, Luo Z, He Y (2015). Genetic analysis of echovirus 11 isolated from patients with viral encephalitis in Longyan, China. Bing Du Xue Bao.

[60] Yarmolskaya MS, Shumilina EY, Ivanova OE, Drexler JF, Lukashev AN (2015). Molecular epidemiology of echoviruses 11 and 30 in Russia: different properties of genotypes within an enterovirus serotype. Infect Genet Evol.

