# Supplementary material for: Analysis of enterovirus genotypes in the cerebrospinal fluid of children associated with aseptic meningitis in Liaocheng, China, from 2018 to 2019
Source: BMC Infect Dis. 2021 May 1;21:405. doi: 10.1186/s12879-021-06112-9 (PMC8088645; doi:10.1186/s12879-021-06112-9)
Supplement: Supplementary file 1 — Additional file 1. The sequences of EVs in this study. [file 12879_2021_6112_MOESM1_ESM.doc]

**The sequences of EVs in this study.**

| **Sample name** | **GenBank accession number** | **Genotype** | **Month/Year of isolation** | **Sequences** | **Clinical diagnosis** |
| --- | --- | --- | --- | --- | --- |
| C1/LC/CHN/2018 | MT646123 | E30 | 04/2018 | VP1 | AM |
| C2/LC/CHN/2018 | MT950626 | E18 | 06/2018 | VP1 | AM |
| C3/LC/CHN/2018 | MT950627 | E18 | 06/2018 | VP1 | AM |
| C4/LC/CHN/2018 | MT950628 | E18 | 06/2018 | VP1 | AM |
| C5/LC/CHN/2018 | MT950616 | E11 | 06/2018 | VP1 | AM |
| C6/LC/CHN/2018 | MT950629 | E18 | 07/2018 | VP1 | AM |
| C7/LC/CHN/2018 | MT901934 | CVB5 | 07/2018 | VP1 | AM |
| C8/LC/CHN/2018 | MT901935 | CVB5 | 07/2018 | VP1 | AM |
| C9/LC/CHN/2018 | MT950617 | E11 | 07/2018 | VP1 | AM |
| C10/LC/CHN/2018 | MT950630 | E18 | 07/2018 | VP1 | AM |
| C11/LC/CHN/2018 | MT950618 | E11 | 07/2018 | VP1 | AM |
| C12/LC/CHN/2018 | MT950619 | E11 | 07/2018 | VP1 | AM |
| C13/LC/CHN/2018 | MT901936 | CVB5 | 07/2018 | VP1 | AM |
| C14/LC/CHN/2018 | MT950620 | E11 | 07/2018 | VP1 | AM |
| C15/LC/CHN/2018 | MT950631 | E18 | 07/2018 | VP1 | AM |
| C16/LC/CHN/2018 | MT950621 | E11 | 07/2018 | VP1 | AM |
| C17/LC/CHN/2018 | MT950599 | E6 | 07/2018 | VP1 | AM |
| C18/LC/CHN/2018 | MT950632 | E18 | 07/2018 | VP1 | AM |
| C19/LC/CHN/2018 | MT950622 | E11 | 07/2018 | VP1 | AM |
| C20/LC/CHN/2018 | MT950623 | E11 | 07/2018 | VP1 | AM |
| C21/LC/CHN/2018 | MT950633 | E18 | 07/2018 | VP1 | AM |
| C22/LC/CHN/2018 | MT950634 | E18 | 07/2018 | VP1 | AM |
| C23/LC/CHN/2018 | MT950600 | E6 | 07/2018 | VP1 | AM |
| C24/LC/CHN/2018 | MT950635 | E18 | 07/2018 | VP1 | AM |
| C25/LC/CHN/2018 | MT901937 | CVB5 | 07/2018 | VP1 | AM |
| C26/LC/CHN/2018 | MT950624 | E11 | 07/2018 | VP1 | AM |
| C27/LC/CHN/2018 | MT950625 | E11 | 07/2018 | VP1 | AM |
| C28/LC/CHN/2018 | MT950636 | E18 | 07/2018 | VP1 | AM |
| C29/LC/CHN/2018 | MT646124 | E20 | 08/2018 | VP1 | AM |
| C30/LC/CHN/2018 | MT950601 | E6 | 08/2018 | VP1 | AM |
| C31/LC/CHN/2018 | MT950602 | E6 | 08/2018 | VP1 | AM |
| C32/LC/CHN/2018 | MT950603 | E6 | 08/2018 | VP1 | AM |
| C33/LC/CHN/2018 | MT901938 | CVB5 | 08/2018 | VP1 | AM |
| C34/LC/CHN/2018 | MT950604 | E6 | 08/2018 | VP1 | AM |
| C35/LC/CHN/2018 | MT950605 | E6 | 08/2018 | VP1 | AM |
| C36/LC/CHN/2018 | MT901939 | CVB5 | 09/2018 | VP1 | AM |
| C37/LC/CHN/2018 | MT950606 | E6 | 09/2018 | VP1 | AM |
| C38/LC/CHN/2018 | MT950607 | E6 | 09/2018 | VP1 | AM |
| C39/LC/CHN/2018 | MT646125 | CVA9 | 09/2018 | VP1 | AM |
| C40/LC/CHN/2018 | MT901940 | CVB5 | 09/2018 | VP1 | AM |
| C41/LC/CHN/2018 | MT950608 | E6 | 09/2018 | VP1 | AM |
| C42/LC/CHN/2018 | MT950609 | E6 | 10/2018 | VP1 | AM |
| C43/LC/CHN/2018 | MT901941 | CVB5 | 10/2018 | VP1 | AM |
| C44/LC/CHN/2018 | MT950610 | E6 | 10/2018 | VP1 | AM |
| C45/LC/CHN/2018 | MT950611 | E6 | 10/2018 | VP1 | AM |
| C46/LC/CHN/2018 | MT950612 | E6 | 10/2018 | VP1 | AM |
| C47/LC/CHN/2018 | MT950613 | E6 | 10/2018 | VP1 | AM |
| C48/LC/CHN/2018 | MT950614 | E6 | 10/2018 | VP1 | AM |
| C49/LC/CHN/2018 | MT950615 | E6 | 11/2018 | VP1 | AM |
| C50/LC/CHN/2018 | MT901942 | CVB5 | 11/2018 | VP1 | AM |
| C1/LC/CHN/2019 | MT950542 | CVA9 | 05/2019 | VP1 | AM |
| C2/LC/CHN/2019 | MT950543 | E30 | 06/2019 | VP1 | AM |
| C3/LC/CHN/2019 | MT950544 | E18 | 06/2019 | VP1 | AM |
| C4/LC/CHN/2019 | MT950545 | CVA9 | 06/2019 | VP1 | AM |
| C5/LC/CHN/2019 | MT950546 | E18 | 06/2019 | VP1 | AM |
| C6/LC/CHN/2019 | MT950547 | E11 | 06/2019 | VP1 | AM |
| C7/LC/CHN/2019 | MT950548 | E30 | 06/2019 | VP1 | AM |
| C8/LC/CHN/2019 | MT950549 | E18 | 06/2019 | VP1 | AM |
| C9/LC/CHN/2019 | MT950550 | E18 | 07/2019 | VP1 | AM |
| C10/LC/CHN/2019 | MT950551 | E30 | 07/2019 | VP1 | AM |
| C11/LC/CHN/2019 | MT950552 | E18 | 07/2019 | VP1 | AM |
| C12/LC/CHN/2019 | MT950553 | E18 | 07/2019 | VP1 | AM |
| C13/LC/CHN/2019 | MT950554 | E30 | 07/2019 | VP1 | AM |
| C14/LC/CHN/2019 | MT950555 | E5 | 07/2019 | VP1 | AM |
| C15/LC/CHN/2019 | MT950556 | E18 | 07/2019 | VP1 | AM |
| C16/LC/CHN/2019 | MT950557 | CVA9 | 07/2019 | VP1 | AM |
| C17/LC/CHN/2019 | MT950558 | E18 | 07/2019 | VP1 | AM |
| C18/LC/CHN/2019 | MT950559 | E18 | 08/2019 | VP1 | AM |
| C19/LC/CHN/2019 | MT950560 | E30 | 08/2019 | VP1 | AM |
| C20/LC/CHN/2019 | MT950561 | E5 | 08/2019 | VP1 | AM |
| C21/LC/CHN/2019 | MT950562 | E18 | 08/2019 | VP1 | AM |
| C22/LC/CHN/2019 | MT950563 | E18 | 08/2019 | VP1 | AM |
| C23/LC/CHN/2019 | MT950564 | CVA9 | 08/2019 | VP1 | AM |
| C24/LC/CHN/2019 | MT950565 | E5 | 08/2019 | VP1 | AM |
| C25/LC/CHN/2019 | MT950566 | E18 | 08/2019 | VP1 | AM |
| C26/LC/CHN/2019 | MT950567 | E11 | 08/2019 | VP1 | AM |
| C27/LC/CHN/2019 | MT950568 | E18 | 08/2019 | VP1 | AM |
| C28/LC/CHN/2019 | MT950569 | CVA9 | 08/2019 | VP1 | AM |
| C29/LC/CHN/2019 | MT950570 | E18 | 08/2019 | VP1 | AM |
| C30/LC/CHN/2019 | MT950571 | E18 | 08/2019 | VP1 | AM |
| C31/LC/CHN/2019 | MT950572 | E18 | 08/2019 | VP1 | AM |
| C32/LC/CHN/2019 | MT950573 | E18 | 08/2019 | VP1 | AM |
| C33/LC/CHN/2019 | MT950574 | E18 | 09/2019 | VP1 | AM |
| C34/LC/CHN/2019 | MT950575 | E5 | 09/2019 | VP1 | AM |
| C35/LC/CHN/2019 | MT950576 | E18 | 09/2019 | VP1 | AM |
| C36/LC/CHN/2019 | MT950577 | E18 | 09/2019 | VP1 | AM |
| C37/LC/CHN/2019 | MT950578 | E18 | 09/2019 | VP1 | AM |
| C38/LC/CHN/2019 | MT950579 | CVA9 | 09/2019 | VP1 | AM |
| C39/LC/CHN/2019 | MT950580 | E18 | 09/2019 | VP1 | AM |
| C40/LC/CHN/2019 | MT950581 | E18 | 09/2019 | VP1 | AM |
| C41/LC/CHN/2019 | MT950582 | E18 | 09/2019 | VP1 | AM |
| C42/LC/CHN/2019 | MT950583 | E18 | 09/2019 | VP1 | AM |
| C43/LC/CHN/2019 | MT950584 | E18 | 09/2019 | VP1 | AM |
| C44/LC/CHN/2019 | MT950585 | E18 | 09/2019 | VP1 | AM |
| C45/LC/CHN/2019 | MT950586 | E18 | 09/2019 | VP1 | AM |
| C46/LC/CHN/2019 | MT950587 | E18 | 09/2019 | VP1 | AM |
| C47/LC/CHN/2019 | MT950588 | E18 | 09/2019 | VP1 | AM |
| C48/LC/CHN/2019 | MT950589 | E5 | 09/2019 | VP1 | AM |
| C49/LC/CHN/2019 | MT950590 | E18 | 09/2019 | VP1 | AM |
| C50/LC/CHN/2019 | MT950591 | E30 | 09/2019 | VP1 | AM |
| C51/LC/CHN/2019 | MT950592 | E18 | 10/2019 | VP1 | AM |
| C52/LC/CHN/2019 | MT950593 | E18 | 10/2019 | VP1 | AM |
| C53/LC/CHN/2019 | MT950594 | E18 | 10/2019 | VP1 | AM |
| C54/LC/CHN/2019 | MT950595 | E18 | 10/2019 | VP1 | AM |
| C55/LC/CHN/2019 | MT950596 | E18 | 10/2019 | VP1 | AM |
| C56/LC/CHN/2019 | MT950597 | E18 | 11/2019 | VP1 | AM |
| C57/LC/CHN/2019 | MT950598 | E18 | 11/2019 | VP1 | AM |
